# Editorial: Mental Disorders Associated With Neurological Diseases

**DOI:** 10.3389/fpsyt.2020.00196

**Published:** 2020-03-24

**Authors:** Yi Yang, Chunxue Wang, Yutao Xiang, Jun Lu, Thomas Penzel

**Affiliations:** ^1^Department of Neurology, The First Hospital of Jilin University, Changchun, China; ^2^Department of Neurology, Clinical Trial and Research Center for Stroke, The First Hospital of Jilin University, Changchun, China; ^3^Department of Neurology, Beijing Tiantan Hospital, Capital Medical University, Beijing, China; ^4^Faculty of Health Sciences, University of Macau, Macao, China; ^5^Department of Neurology, Beth Israel Deaconess Medical Center, Harvard Medical School, Boston, MA, United States; ^6^Sleep Medicine Center, Charite University Hospital, Berlin, Germany; ^7^Saratov State University, Saratov, Russia

**Keywords:** mental disorders, post-stroke depression, cognitive impairment, depression, post-stroke anxiety, Parkinson's disease

Mental disorders are important comorbidities of nervous system diseases and they have a lot in common in risk factors and pathogenesis. However, mental disorders can be easily neglected by neurologists. The mechanisms underlying the association of mental disorders with neurological disease are largely unclear. This Research Topic provides a collection of research into post-stroke depression and anxiety, cognitive impairment, and depression in Creutzfeldt-Jakob and Parkinson's disease. Although the present research collection cannot cover the whole range of advancements in the field, it highlights certain key findings regarding mental disorders associated with neurological diseases and we hope that this will inspire further interest and new research efforts in this exciting area.

Depression is a global chronic medical illness that leads to low mood, loss of interest, change in appetite, insomnia, and neurocognitive dysfunction. Despite the prevalence of depression, there are still many aspects to be explored and understood. An increasing prevalence of late-life depression has been identified, the mechanisms of which remain unclear. Previous studies demonstrated that iron deposition was related to the severity of symptoms in patients with depression. Zhang et al. investigated the role of iron deposits in depression among older adults and found that iron deposits in the thalamus was an independent factor relating to depressive symptoms. This new finding may inform future studies into the underlying pathophysiological mechanisms of depression.

Cerebral autoregulation was initially considered as an intrinsic protective mechanism of the brain, which ensures relatively constant cerebral blood flow despite fluctuations in arterial blood pressure or cerebral perfusion pressure. The impairment of cerebral autoregulation has been reported to be a feature of several diseases, including cerebral stroke, and Alzheimer's disease. Luo et al. observed that cerebral autoregulation was compromised in patients with depression and negatively correlated with the depression score. Though the mechanism is still unknown, improving cerebral autoregulation could be a potential therapeutic approach to treating the neurological symptoms of depression.

Depressive disturbances are common in patients with Parkinson's disease, but the neurochemical changes that occur in these cases are still unknown. In order to address this, Lian et al. investigated clinical features and neurochemical changes in patients with Parkinson's disease. The authors report that a high proportion of patients with Parkinson's disease had depression. Motor symptoms, postural instability, gait difficulty, anxiety, and fatigue are the significant influencing factors in cases of Parkinson's disease with depression. Moreover, dopamine may play a more important role in Parkinson's disease with depression compared to 5-HT. In another study, Zhu et al., observed that high concentrations of dopamine may cause the high incidence of restless leg syndrome (RLS) in Parkinson's disease patients, which was accompanied by anxiety, depression, insomnia, and other mental health symptoms. This finding highlights the importance of monitoring such symptoms in the clinical management of patients with Parkinson's disease.

Post-stroke depression, the most common psychiatric implication of stroke, negatively impacts patients' rehabilitation results, cognitive function, and quality of life. In this Research Topic, Huang, Zhao et al. explore the potential interaction between depressive symptoms and cognitive impairment after stroke and found remitters of post-stroke depression had more significant cognitive improvements than non-remitters. Therefore, early recognition and intervention for potential depression after stroke is of great importance. This study also identified predictors of remission in patients with early-onset post-stroke depression, which included neurological impairment, major life events, major medical comorbidities, and frontal lobe lesion at baseline. A review in this Research Topic by Wang, Shi et al. provides a comprehensive overview of etiologies of post-stroke depression. In their article, the authors identify several factors related to the pathogenesis of post-stroke depression, including monoamine neurotransmitter change, inflammation, the hypothalamus-pituitary-adrenal axis and the hypothalamus-pituitary-thyroid axis, glial cells (astrocytes and microglia), vitamin D levels, homocysteine levels, neural network dysfunction, genetic background, and social psychological mechanisms.

Focusing on depression after intracerebral hemorrhage, Wu et al. provide a detailed review about the pathophysiology of depression after intracerebral hemorrhage, including inflammation, oxidative stress, apoptosis, and autophagy. This overview is accompanied by an in-depth exploration of the associated signaling pathways.

Recently, there has been an international focus on research into inflammation and post-stroke depression. Fang et al. produced a comprehensive review summarizing how neuroinflammation affects stroke rehabilitation and post-stroke depression, potentially offering new therapeutic targets for stroke and post-stroke depression. Contributing also to the topic of post-stroke depression, Wang, Wang et al. share their research into the association between post-stroke depression, aphasia and physical independence, in stroke patients in China at a 3-months follow-up. The authors found the incidence of post-stroke depression was independently associated with physical dependence.

Another prevalent mental disorder after a stroke is anxiety. However, studies investigating the effects of post-stroke anxiety on functional status are very limited. The article by Li et al. describes that severity of post-stroke anxiety in the acute stage was a significant indicator for daily living and stroke-specific quality of life.

In this Research Topic, Liang et al. report on a case of sporadic Creutzfeldt-Jakob disease with depression. They found that the patient's condition worsened after using antidepressants. This finding was followed by a systematic survey, which showed that survival period was associated with the type of antidepressant used (especially serotonin and noradrenaline reuptake inhibitors).

Insomnia is a highly prevalent symptom in patients with mental disorders. Exploring the common and different brain mechanisms underlying such symptoms may help refine existing treatments. Yu et al. found that the interaction of depression and insomnia was associated with decreased gray matter volume in the right orbitofrontal cortex. This finding provides new insights into the mechanisms underlying the comorbidity of insomnia and depression. The comorbidity of insomnia and anxiety disorders is also worthy of further exploration. Another study into insomnia in this Research Topic is from Huang, Zhan et al., who reveal that cortical excitability in patients with generalized anxiety disorder comorbid with insomnia is modulated by insomnia. The authors examined the recovery functions of median nerve somatosensory evoked potentials, thus shedding light on the underlying neurobiological correlates of the effects of insomnia on generalized anxiety disorder.

Cognitive impairment seems to mark a high-risk population for developing dementia and plays a crucial role in the course of mental disability. The pathophysiology of it is a field where much work is yet to be done. The contribution from Wei et al. addresses a surrogate marker (the peak width of skeletonized mean diffusivity) for cognitive impairment in cerebral white matter lesions patients, which provides new insights into the pathophysiology of cognitive impairment in these patients. A study in this Research Topic by Deng et al. found that patients with severe vertebra-basilar stenosis showed a decline in cognitive ability, and that chronic posterior circulation hypoperfusion was an independent risk factor for cognitive impairment. The cerebral venous system also plays an important part in the progress of cognitive impairment. Compression and stenosis of the draining veins have been reported to be linked with transient global amnesia (a specific kind of cognitive impairment) via magnetic resonance imaging studies. Using ultrasound examination, Han et al. further confirmed a decrease in the total flow volume of the vertebral and internal jugular veins in patients with transient global amnesia. In addition, internal jugular vein drainage was relatively compromised during the Valsalva maneuver (an activity that can trigger transient global amnesia). To clarify the mechanisms involved in dementia, Zhou et al. performed a meta-analysis of the association between cortical superficial siderosis and dementia. The authors found that pre-existing cortical superficial siderosis could be a candidate imaging indicator for Alzheimer's disease. Another interesting study in this Research Topic is from Yin et al., who found that cerebral blood flow damage in white matter is associated with global cognitive dysfunction in CADASIL (cerebral autosomal dominant arteriopathy with subcortical infarcts and leukoencephalopathy). Moreover, this exploratory study found cerebral blood flow was more strongly associated with global cognitive function than mean diffusivity and that this could be a biomarker used to monitor alterations of global cognitive function in CADASIL.

Finally, the editors would like to acknowledge the authors who contributed to this Research Topic. Their honest efforts and hard work are truly admirable. The editors hope that papers comprising this Research Topic will inspire significant progress in the field of mental disorders associated with neurological diseases.

## Author Contributions

YY: drafted the manuscript. CW, YX, JL, and TP: revised the manuscript. All authors read and approved the final manuscript.

### Conflict of Interest

The authors declare that the research was conducted in the absence of any commercial or financial relationships that could be construed as a potential conflict of interest.

## Funding

This article was supported by the National Key R&D Program of China (2016YFC1301600) to YY, and the RF Government grant No 075-15-2019-1885 to TP.

